# Correction to: Association of cord blood asprosin concentration with atherogenic lipid profle and anthropometric indices

**DOI:** 10.1186/s13098-022-00867-0

**Published:** 2022-07-15

**Authors:** Hanan Khudhair Hussein, Nassrin Malik Aubead, Hamzah H. Kzar, Yasir Salam Karim, Ali H. Amin, Moaed E. Al-Gazally, Tousief Irshad Ahmed, Mohammed Abed Jawad, Ali Thaeer Hammid, Abduladheem Turki Jalil, Yasser Fakri Mustafa, Marwan Mahmood Saleh, Hafez Heydari

**Affiliations:** 1 College of Medicine, University of Babylon, Babil, Iraq; 2 Department of Obstetrics and Gynecology, Hammurabi College of Medicine, University of Babylon, Babil, Iraq; 3 Veterinary Medicine College, Al-Qasim Green University, Al-Qasim, Iraq; 4 Al-Manara College For Medical Sciences, Maysan, Iraq; 5 Deanship of Scientific Research, Umm Al-Qura University, Makkah, Saudi Arabia; 6 Zoology Department, Faculty of Science, Mansoura University, Mansoura, Egypt; 7 College of Medicine, University of Al-Ameed, Karbala, Iraq; 8 Department of Biochemistry, SKIMS, Srinagar, J&K India; 9 Al-Nisour University College, Baghdad, Iraq; 10 Computer Engineering Techniques, Faculty of Information Technology, Imam Ja’afar Al-Sadiq University, Baghdad, Iraq; 11 Faculty of Biology and Ecology, Yanka Kupala State University of Grodno, 230023 Grodno, Belarus; 12 College of Technical Engineering, The Islamic University, Najaf, Iraq; 13 Department of Pharmaceutical Chemistry, College of Pharmacy, University of Mosul, Mosul, 41001 Iraq; 14 Department of Biophysics, College of Applied Sciences, University Of Anbar, Ramadi, Iraq; 15 Non-Communicable Diseases Research Center, Sabzevar University of Medical Sciences, Sabzevar, Iran

## Correction to: Diabetology & Metabolic Syndrome (2022) 14:74 https://doi.org/10.1186/s13098-022-00844-7

Following publication of the original article [1], the name of second author has been changed from Malik Aubead to Nassrin Malik Aubead.

The original article has been revised.

## References

[1] Hussein HK, Aubead NM, Kzar HH, Karim YS, Amin AH, Al-Gazally ME, Ahmed TI, Jawad MA, Hammid AT, Jalil AT, Mustafa YF (2022). Association of cord blood asprosin concentration with atherogenic lipid profile and anthropometric indices. Diabetol Metab Syndr.

